# Relationship of maxillary third molar root to the maxillary sinus wall: A cone-beam computed tomography (CBCT) based study

**DOI:** 10.34172/joddd.2023.30484

**Published:** 2023-04-03

**Authors:** Humayun Kaleem Siddiqui, Aysha Arif, Kanza Ghauri, Anum Aijaz, Farhan Raza Khan

**Affiliations:** Department of Surgery, Aga Khan University Hospital, Karachi, Pakistan

**Keywords:** Maxillary third molars, Maxillary sinus, CBCT imaging

## Abstract

**Background.:**

The relationship of the root of the maxillary third molars and the maxillary sinus (MS) is an important predictor of the anticipated difficulty in extraction. The aim of this study was to assess the location of maxillary third molars to the inferior wall of the MS in a sample of Pakistani population evaluated using cone-beam computed tomography (CBCT) imaging and to assess if age or gender has any influence on third molar to MS distance.

**Methods.:**

The CBCT scans of adult patients, carried out keeping image volume at 8 cm×8 cm, and the voxel size 0.2 and 0.1 mm. Images retrieved from the hospital database were included in the study. The relationship of root apices of maxillary third molar with the MS was assessed according to the vertical, horizontal and Winter’s classification. Descriptive statistics, *t* test and chi-square test of association were applied.

**Results.:**

CBCT scans of 93 patients, 56 males and 37 females were evaluated. The mean age was 41.12±17.13 years. The mean distance of third molar roots to the MS wall was 2.38±1.54 mm for males and 1.86±1.04 mm for females, on the left and 2.67±1.81 mm for males and 2.58±1.54 mm in females, on the right side. Independent sample *t* test showed that there was no significant difference for third molar to sinus wall distance in the two genders. No significant difference was found between the two sides.

**Conclusion.:**

In a sub-population of Pakistani adults, the mean distance between the roots of the upper third molar and MS wall is around 2 mm. Only 5% males and 8% females had their upper third molars roots protruding into the MS.

## Introduction

The maxillary sinus (MS) starts developing soon after birth and completes its growth in the second decade of life.^1^ The MS extension in adults is variable. Its floor is formed by the alveolar process of the maxilla.^2^ The roots of molars, premolars and even canine may extend into the sinus forming elevated projections, referred in literature as “Hillhocks”.^3^ The thickness of bone between the root apices and the MS also varies.^2^ The amount of pneumatization and ethnic variation in the anatomy of the mid face affects the relationship of maxillary third molar root apices with the MS.^4,5^ Infection affecting molar and premolars (periodontal or periapical), may involve the MS causing sinusitis. Other procedures such as extraction of teeth can lead to perforation or root displacement into the MS or formation of an oro-antral fistula.^6,7^

Preoperative assessment of the difficulty of upper third molar extraction is an important task.^8^ The extraction of upper third molars requires considerable planning and skills, from diagnosis to intra-operative and post-operative management.^9^ Several assessment models have been suggested in the literature but they were based on surgical difficulty encountered with management of mandibular third molars; the maxillary third molars have been investigated considerably less.^10,11^ Among the few predictors that have been reported in the literature are, the amount of buccal tissue, mouth opening greater than 45 mm, number and root positioning.^12^ The relationship of the upper third molars and MS has been documented as a statistically significant predictor of the surgical difficulty.^12^ Most of the published literature on the maxillary third molars are actually case reports. These reports document the occurrence of complications with the MS while extracting the third molars and how to manage the complications. There is scarcity of literature on the assessment of surgical difficulty to prevent the complications.^13^ This has led the dentists to underestimate the complications with the maxillary third molar surgery. De Carvalho et al reported that around 56% of all upper third molar extraction were difficult and the relationship of third molar to MS was among the five predictors that influenced the surgical difficulty of the extraction. Therefore, it is important to identify the difficult cases, make appropriate referrals and obtain informed consent from the patient.^14^

For preoperative assessment of the relationship of the MS floor with the posterior teeth, clinicians used to employ periapical and panoramic radiographs. With the availability of cone-beam computed tomography (CBCT), the shortcomings of the two-dimensional radiography such as magnification, distortion and superimposition are now largely overcome. The advantage of CBCT is that it provides three-dimensional, uniform multi-planar images with low magnification.^15^ Presently, CBCT has now become the standard imaging modality for the evaluation of MS, since both the soft tissues and the bone, can be viewed in multiple images in thin sections.^16^

The relationship of upper third molars root and MS is also affected by age, gender, extent of pneumatization and ethnic variation in the anatomy of the mid-face.^17^ There is scarcity of studies on the relationship of MS and the roots of the upper third molar in Pakistani population. The present study is aimed to assess the relationship of upper third molars with the inferior wall of the MS in a sample of Pakistani population using CBCT imaging.

## Methods

 The ethical exemption for this study was obtained from the ethics committee (reference # 2018-0368-702) of the Aga Khan University, Karachi, Pakistan. The CBCT scans from November 2017 onwards were included. The demographic information (age and gender) of the patients were obtained by reviewing the medical record files. Patients with no history of dental extractions, or surgeries involving MS, or, orthodontic treatment, or any other intervention that could affect the position of the maxillary posterior teeth were selected. Subjects with any craniofacial anomaly, pathology of dento-alveolar region, or having poor-quality CBCT images, or with incomplete root formation, or loss of the adjacent second molar, were excluded.

 The CBCT scans were carried out using the ORTHOPHOSXG 3D Ready/CEPH (SIRONA) operating at 60 kV-90 kV/3 mA-6 mA, image volume 8 cm × 8 cm, and the voxel size was 0.2 and 0.1 mm, scanning time is 14 seconds and exposure time is 2-5 seconds. The images were saved using SIDEXIS software. The CBCT images were viewed on a monitor HP Elite Display, with the resolution of 1920 × 1080 @ 60 Hz, Contrast static: 1000:1; Dynamic: 5 000 000:1. The measurements were made by the primary investigator.

 The CBCT images of the maxilla were examined in three viewing planes: tangential, axial, and cross-sectional view. The measurements were obtained in two views: tangential and cross-sectional.

The vertical relationship between the roots of the upper third molars and the sinus floor was rated on following rating^18,19^:

- Type I: The MS floor was located at the top of the level connecting the buccal and palatal root tips,
- Type II: The MS floor located at a lower level connecting the buccal and palatal root tips without an apical protrusion over MS floor,
- Type III: Buccal root tips protruded into the MS floor,
- Type IV: Palatal root tip protruded into the MS floor,
- Type V: Buccal and palatal root tips protruded into the MS floor.

For the horizontal relationship, following rating was employed:

- Type 1: The MS floor was protruded more toward the buccal side than toward the buccal root
- Type 2: The MS floor was protruded between the buccal and palatal roots
- Type 3: The MS floor was protruded more toward the palatal side than toward the palatal root.

The axial relationship of tooth was assessed using Winters classification. It is the relationship between the long axis of the second molar and the long axis of the third molar. It has six categories of angulations (vertical, horizontal, mesio-angular, bucco-palatal, disto-angular, and others). All the length measurements were recorded on a proforma in a tabulated form and then entered into the SPSS version 23.0 (IBM, USA) for data analysis.

## Results

CBCT scans of 93 patients, 56 males and 37 females were assessed. The mean age was 41.12 ± 17.13 years (41.05 ± 17.79 for males, 42.30 + 14.33 for females). The mean distance of third molar roots to the MS wall was 2.38 ± 1.54 mm for males, and 1.86 ± 1.04 mm for females (Table 1). Independent sample *t *test showed that there was no significant difference between the genders for third molar roots to the MS wall distance. No significant difference was found between the two sides of the jaw (Table 2). No association between the gender and the type of tooth classification (horizontal, vertical or Winter’s) could be seen (Table 3).

**Table 1:** Descriptive Statistics of distance between maxillary third molar and maxillary sinus wall

**Side **	**Gender**	**n**	**Mean (mm)**	**SD (mm)**	***P *** value*
Left	Male	43	2.38	1.54	0.10
	Female	26	1.86	1.04	
Right	Male	48	2.67	1.81	0.83
	Female	24	2.58	1.54	

* Independent samples *t* test was applied. A *P* value ≤ 0.05 was considered as statistically significant.

**Table 2:** Difference between right and left side of the maxilla for third molar to sinus wall distance

**Side**	**n**	**Mean (mm)**	**SD (mm)**	***P *** value*
Left	50	2.16	1.22	0.59
Right	50	2.27	1.55	

* Paired sample *t* test was applied. A* P* value ≤ 0.05 was considered as statistically significant.

**Table 3:** Association of gender with third molar and maxillary sinus relationship

**Classification**	**Type**	**Males**		**Females**		***P *** value*
		**No.**	**(%)**	**No.**	**(%)**	
Vertical classification	1	28	50.0	17	45.9	NS
	2	25	44.7	17	45.9	
	3	2	3.6	3	8.2	
	4	1	1.7	0	0	
Total		56	100%	37	100%	
Horizontal classification	1	30	54.5	19	51.3	NS
	2	23	40.0	17	46.0	
	3	3	5.5	1	2.7	
Total		56	100%	37	100%	
Winter’s classification	1	52	92.8	35	94.5	NS
	2	3	5.4	2	5.5	
	3	1	1.8	0	0	
Total		56	100%	37	100%	

*Chi-square/Fisher Exact test were employed. A *P* value ≤ 0.05 was considered as statistically significant. NS: not significant.

## Discussion

The relationship of the upper third molar with the MS is of prime importance when planning surgery or extraction in the area, which must be aided by appropriate radiograph.^20^ Knowledge of the anatomy also aids to allow efficient and safe removal of bone during extractions.^21^ Conventionally, panoramic radiographs have been used to evaluate it but factors such as superposition of structures, unwanted magnification, and lack of cross-sectional views are the major drawbacks of the two-dimensional imaging.^22^ Significant differences have been reported between the measurement obtained from panoramic radiograph and CBCT. The CBCT has become a reliable imaging modality for the evaluation of upper third molars and MS walls.^20^

Considering the vertical classification, type I and II are the most frequently observed orientation in our study (48.3% and 45.1%, respectively). In most of the CBCT images evaluated in the study, it was observed that the MS floor was located superior to the upper third molar root apices, or at a lower level but without any root tips protruding into the MS. The pattern of occurrence of types mentioned is similar in both genders. Similar pattern of occurrence was seen in few other studies. Yurdabakan et al^23^ reported 43.5% frequency in Nigerian population for type I vertical relationship, however type II frequency was only 16.1%. Study on Turkish population, by Kilic et al^20^ reported the most recurrent to be type I.

The least encountered vertical classification in our sample was, type III and IV. Yurdabakan et al reports similar findings with type III occurrence 18.5% and type IV only 1%.^23^ However another study in Turkish population, by Demirtas and Harorli^18^ reported the most occurring type of vertical class to be type III. The differences that occur in the results probably attributed to the racial traits, difference in the sample size and the imaging techniques employed.

The incidence of deep protrusion of the upper third molar roots were extremely rare in the present study which can lead to the inference that the potential of an oro-antral fistula developing would be low, provided correct surgical technique is used. A study reports existence of sexual dimorphism in development of oro-antral fistula after extractions.^24^ This may account to the increased incidence of traumatic extraction in males.^25^ Existing literature reports 0.8% incidence of oro-antral fistula after maxillary third molar extraction, with fistulas of less than 2 mm healing spontaneously, larger require surgical intervention.^26,27^ These fistulas may also develop after removal of cysts from the maxilla, tumors, or dento-facial trauma, or implant surgery, infection or of iatrogenic origin. A study suggests increased incidence of fistula formation in ages between 30 and 60 years.^28^

For the horizontal classification, type I was the most common relationship in our study (54.5%). Pattern of occurrence in both genders was similar. Yurdabakan et al^23^ reported the most frequently occurring relationship was type II (59.3%), as MS floor was found to be protruded between the buccal and palatal roots. Demirtas and Harorli^18^ on the other hand, reported type II as the most occurring class, i.e., sinus was present between the roots.

For Winter’s classification, type I (bucco-palatal) was predominantly occurring type in both genders in our study and the least occurring type of relationship was mesio-angular impaction. Yurdabakan et al reports vertical as the most recurring type of angulation of the maxillary third molar in Nigerian population (94.3%) and least occurring type was horizontal angulation of impaction. Studies conducted in Iranian population and French population have reported vertical angulation to be the most commonly occurring angulation which contrasts with the findings of our study. Kruger et al. reported mesio-angular to be the most commonly occurring angulation of maxillary third molars among New Zealanders.^29^

## Conclusion

 In a sub-population of Pakistani adults, the mean distance between the roots of the upper third molar and MS wall is around 2 mm. Only 5% males and 8% females had their upper third molars roots protruding into the MS. Anatomical differences require three-dimensional pre-procedural planning mandatory for the surgical procedures planned in the area.

## Acknowledgments

 We are deeply thankful for the radiology archives manager of the dental section, AKUH for providing us the CBCT data for our study.

## Competing Interests

 None.

## Ethical Approval

 The ethical exemption (reference # 2018-0368-702) for this study was obtained from the ethics committee of the Aga Khan University, Karachi, Pakistan.

## Funding

 None.
